# Assessment of Unfrozen Water Content in Copper Bentonites Using the ^1^H NMR Technique: Optimization, the Method’s Limitation, and Comparative Analysis with DSC

**DOI:** 10.3390/ma16247577

**Published:** 2023-12-09

**Authors:** Edyta Nartowska, Maria Kanuchova, Ľubica Kozáková

**Affiliations:** 1 Faculty of Environmental Engineering, Geomatics and Renewable Energy, Kielce University of Technology, al. 1000-lecia PP 7, 25-314 Kielce, Poland; 2 Faculty of Mining, Ecology, Process Control and Geotechnologies, Technical University of Kosice, Letna 9, 042 00 Kosice, Slovakia; maria.kanuchova@tuke.sk (M.K.); lubica.kozakova@tuke.sk (Ľ.K.)

**Keywords:** NMR, unfrozen water, clays, chemical shift, copper

## Abstract

Studies on changes in unfrozen water content in copper bentonite from Slovakia were conducted using both differential scanning calorimetry (DSC) and nuclear magnetic resonance (NMR) methods. The aims of this study were to 1. optimize the method for determining changes in unfrozen water content using the ^1^H NMR technique in model bentonites based on the DSC results; 2. analyze the relationship between unfrozen water content, as determined via DSC and the optimized NMR technique, and the physicochemical parameters of bentonites; and 3. identify the limitations in determining changes in unfrozen water content using the ^1^H NMR technique in relation to copper-contaminated bentonites. The results obtained using the optimized NMR method applied to the model bentonites correlated well with the DSC results. The unfrozen water content in the Cu-contaminated bentonites was 2–18% lower after NMR compared to the DSC results, likely due to the mobility of copper ions and their paramagnetic properties. Statistically significant differences in unfrozen water content between the DSC and NMR methods were observed, depending on molar concentration, copper ion concentration, and temperature, confirmed via Analysis of Variance (ANOVA). Calorimetric studies are recommended for investigating unfrozen water content changes in contaminated clays. Further NMR research could identify metals influencing free induction decay signals under varying physicochemical conditions.

## 1. Introduction

Bentonites, due to their unique adsorptive properties, have found widespread applications in various fields, such as construction, engineering, environmental protection, medicine, and the food industry [1,2,3]. In the context of environmental protection, bentonite is frequently used as a sealing barrier in landfills and wastewater treatment plants. Its role is to prevent harmful substances from infiltrating into the soil and groundwater [4]. In recent decades, there has been significant interest among researchers in the use of bentonite as an insulating material between canisters for high-level nuclear waste and bedrock [5,6]. In research related to bentonites, especially in the field of environmental protection, a significant challenge is posed by their exposure to potentially toxic metal ions [7]. These ions can significantly alter the physicochemical and microstructural properties of bentonite, a fact confirmed by previous studies [8,9,10,11]. It is worth noting that copper ions, as an example of such metals, influence the unfrozen water content in clayey soils [12,13]. This portion is the amount of water that remains liquid in the soil even at extremely low negative temperatures. Its quantity is closely related to the change in negative temperature and, to a lesser extent, to the physicochemical properties of soils [14]. This liquid water layer plays a crucial role in many material properties, such as stability and reactivity. Understanding unfrozen water content is vital in various fields, ranging from geotechnical engineering, where it affects slope stability and landslide hazards, to agriculture, where controlling this content is essential for the healthy growth of plants [15]. Furthermore, in the context of natural environmental protection, there is a need to analyze its impact on the circulation of groundwater and its influence on ecosystems. Therefore, research on unfrozen water content in bentonites is crucial for a comprehensive understanding of their properties and optimal utilization in different scientific and industrial domains.

Determining changes in the unfrozen water content in bentonites can be conducted using various methods [14,16,17]. Nuclear Magnetic Resonance techniques (NMR) and Differential Scanning Calorimetry techniques (DSC) are among the most widely used electrical and thermal methods for determining unfrozen water content in soils [18,19,20,21,22]. The DSC process involves simultaneously heating a sample and a reference material and then measuring the difference in the amount of heat absorbed or released by the sample compared to the reference material as a function of temperature. The DSC technique is advantageous in this regard due to its relatively quick measurement process and small samples. It helps prevent errors resulting from supercooling and the slow thermal equilibration between the sample and its container as well as the sluggish equilibration between unfrozen water and ice within the sample [14]. Interpreting DSC curves related to unfrozen water can be challenging, requiring experience and knowledge about the specific characteristics of this phenomenon. However, the utility of this method has been confirmed across a wide range of soil materials, including in the study of soils contaminated with copper ions [13,14,18]. The NMR technique is based on the phenomenon of magnetization. Hydrogen nuclei, carrying a positive charge and spinning, create a magnetic moment. When a magnetic field is applied, these magnetic dipoles align in the moment’s direction, resulting in magnetization. The degree of magnetization is directly related to the quantity of protons and inversely related to temperature, following Curie’s law. The frequency at which nuclei align is determined by the strength of the magnetic field and the type of nuclei. A radiofrequency pulse (RF), perpendicular to the primary magnetic field, stimulates nuclei, initiating free induction decay (FID) as they return to equilibrium. The physicochemical nature of clays, including their adsorbed cations, strongly influences the quantity and mobility of unfrozen water [23]. The NMR method allows for real-time monitoring of interactions between soil and a solvent. Therefore, this method holds great potential for aiding in the understanding of processes occurring in soil–water systems [24,25].

The prevailing assumption is that the FID signal originates solely from liquid water given the rapid nature of the FID for ice [14,20]. The final unfrozen water content is calculated based on a formula that relies on three parameters: (i) the value of the FID signal, which is read at a negative temperature; (ii) the natural water content of the sample measured using an independent oven-drying method (with the corresponding value constituting the ratio of water mass to the mass of the soil skeleton expressed as a percentage); and (iii) the adopted reference FID signal value, read at a positive temperature. The value of the reference FID signal is challenging to capture due to the fact that the signal intensity, at positive temperatures, begins to decrease after reaching an unspecified temperature, possibly due to the paramagnetic interaction of ions [19,20,21,22]. In the literature, the formulas for determining changes in unfrozen water content based on FID signals vary in terms of the adopted reference FID signal value: FID_reference_ at 0 °C [19], FID_reference_ at 5 °C [20], and FID_reference_ determined using specific functions [21,22]. Adopting different FID_reference_ values influences the final result regarding the unfrozen water content. It seems essential to optimize the formula corresponding to the studied clays.

The literature lacks information regarding the assessment of unfrozen water content changes in clayey soils contaminated with potentially toxic metal ions. Preliminary studies conducted by the authors of this paper using the NMR technique suggested that NMR might not be suitable for estimating changes in unfrozen water content in clays containing high concentrations of copper ions [12]. The observed unfrozen water content values were disproportionately low [12]. It is possible that in such soil–water systems, the phenomenon of a chemical shift occurs, and water molecules involved in the hydration process of cations are not detected via NMR [26]. The chemical shifts of protons in water can depend on the type and structure of the metal ions with which they form hydration complexes. In NMR interpretation, paramagnetic ions (such as Fe^3+^, Mn^2+^, Ni^2+^, and Cu^2+^) are often considered a complicating factor because they have a substantial impact on relaxation times [27]. Additionally, soils constitute heterogeneous materials, and copper ions interacting with other compounds present in the soil structure can exhibit different magnetic phases. For instance, copper halides from the atacamite group are relatively unstable [27]. This is particularly concerning because they form in bentonites with high concentrations of copper ions [13]. Further research is needed to explain the reason for the observed low unfrozen water content in Cu bentonites determined using the NMR method, taking into account varied concentrations of high copper content in clays. Previous studies were conducted on bentonite with a single high concentration of copper ions, without the presence of minerals from the atacamite group. Considering the diverse nature of copper ions and their ability to form complexes and new mineral phases, it cannot be ruled out that different concentrations of copper ions might have different impacts on the liquid water content in soil at temperatures below 0 °C. It is also essential to compare the obtained results with those obtained using the DSC method. A comprehensive analysis can shed light on the current limitations faced in determining unfrozen water content in copper bentonites using the NMR method, and its integration with DSC techniques presents promising prospects for gaining a more comprehensive understanding of unfrozen water in clay complex systems. 

Therefore, the aims of this work were to (1) determine changes in the intensity of the free induction decay (FID) signal of hydrogen ions in source bentonites containing high concentrations of exchangeable copper cations using the ^1^H NMR technique in the temperature range of −32 °C to +23 °C; (2) optimize the method for determining changes in unfrozen water content using the ^1^H NMR technique in the studied bentonites; (3) compare changes in unfrozen water content determined using the DSC method with the results obtained using the optimized ^1^H NMR technique and do so in accordance with the empirical equations of Akagawa et al. [19], Kruse et al. [20], Li et al. [21], and He et al. [22]; (4) examine the correlation between unfrozen water content determined through DSC and NMR techniques and the molar and copper concentrations, as well as the physicochemical parameters of bentonites; and (5) identify the limitations in determining changes in unfrozen water content using the ^1^H NMR technique in relation to copper-contaminated bentonites.

## 2. Materials and Methods

### 2.1. Materials

Two model clays, Stx-1b (from Gonzales County, TX, USA) and BSvk (from Stara Kremnička—Jelšový potok, Slovakia), were used to study the unfrozen water content in bentonites. Bentonite was tested in its natural state (Stx-1b; BSvk) and in homoionic forms with copper (BSvk Cu). This resulted in four homoionic forms of bentonites, denoted as BSvk 1 M Cu (copper form saturated with 1 mol/dm^3^ copper (II) chloride solutions—Merck, Darmstadt, Germany); BSvk 0.1 M Cu; BSvk 0.25 M Cu; and BSvk 0.5 M Cu (copper forms of Slovak bentonite saturated with copper (II) chloride solutions—Merck, Darmstadt, Germany ranging from 0.1 to 0.5 mol/dm^3^). These bentonites consisted of at least 75% smectite, along with silica minerals and biotite. In cases of bentonite’s contamination with Cu ions at concentrations of 0.1–0.5 M, 3–6% proportions of atacamite (copper hydroxychloride) minerals were present [13]. Table 1 summarizes the physicochemical properties of the clays based on our prior research [13,18].

### 2.2. Methods

#### 2.2.1. Preparation of Homoionic Forms and Properties of Bentonites

Bentonite samples weighing 50 g were poured with 10 L of copper (II) chloride solutions with concentrations of 0.1–0.25–0.5–1 mol/dm^3^ to obtain monoionic forms. After 48 h, the solution was decanted, and this process was repeated three times. The remaining suspension was transferred to membranes permeable to chloride ions and placed in a bucket with constant circulation of distilled water. The rinsing process continued for approximately 35 days until the reaction to Cl^−^ ions, detected using silver nitrate, disappeared. The monoionic bentonite samples were subsequently air-dried and stored in containers. Furthermore, the absence of free chloride ions was confirmed through titration (PN-ISO 9297:1994 [28]).

The metal content was determined using inductively coupled plasma optical emission spectrometry (ICP-OES) after prior extraction of the samples with aqua regia. Prior to conducting the analysis, the instrument underwent calibration using a multi-element standard (Instrument Calibration Standard 2, No. N9301721, PerkinElmer, Waltham, MA, USA).

The inter-packet distances in the bentonites in the plane parallel to the sample surface d001 were determined based on X-ray diffractograms. For the mineralogical study, a Bruker D8 Advance X-ray diffractometer––a Johansson-type monochromator––was used to determine the CuKα1 radiation (l = 1.5406 Å) with the assistance of a LynxEye position-sensitive detector (Bruker devices, Berlin, Germany). The measurements were carried out in 2θ from 4.51° to 70° with 0.02° steps. The applied voltage was 3.540 kV with a 530 mA current.

The clay and silt fraction was analyzed using a HELOS/BF SUCELL laser diffractometer produced by Sympatec GmbH (Clausthal Zellerfeld, Germany). Prior to the analysis, 3 g of clay paste was mixed with 50 mL of distilled water. The clay paste was prepared by saturating bentonite with distilled water to achieve a soft plastic consistency 48 h before the analysis.

The specific surface area, which is valuable for studying clay–water systems, was assessed using the water vapor sorption (WST) method, as outlined by Stępkowska [29].

To be apprised of the detailed methodology, refer to the authors’ previous work [12,13].

#### 2.2.2. NMR Technique

##### NMR Equipment

**Figure 1:**
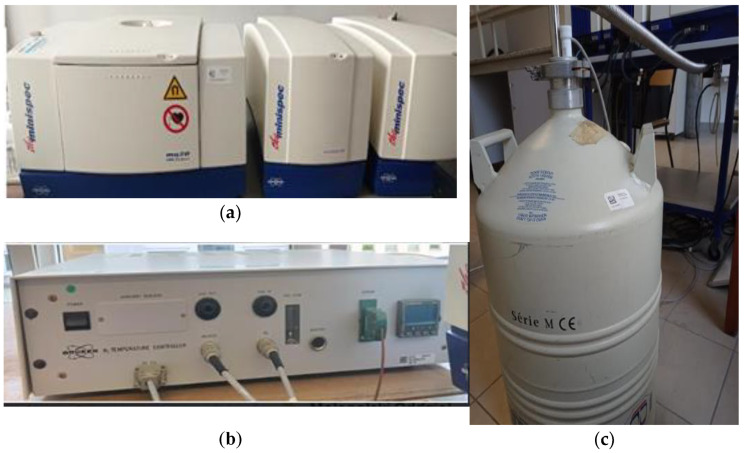
NMR minispec mq20 equipment: (**a**) magnetic, gradient, and electronic units; (**b**) temperature control unit, BVT 3000; (**c**) liquid nitrogen system.

To study changes in the unfrozen water content as a function of temperature, a minispec mq20 apparatus from Bruker with a magnetic field strength of 0.47 T was employed (Figure 1). The use of this apparatus allowed for the freezing and thawing of the sample directly within the research chamber. The temperature of the samples placed in the magnetic unit chamber was controlled via a BVT 3000 (Bruker, Berlin, Germany) temperature control unit with a connected liquid nitrogen system [12]. Prior to commencing the analyses, the system was calibrated. Additionally, a ‘Daily Check’ procedure was performed every 24 h. Running this procedure ensures compliance with Good Laboratory Practice (GLP). Utilizing a certified standard provided by Bruker Corporation (distilled water with 0.5% CuSO_4_ * 5 H_2_O), the correctness of settings such as gain, magnetic fields, detection angles, and pulse lengths was verified.

The NMR signal was induced by radiofrequency pulses at a specific frequency known as the Larmor frequency, which depends on the magnetic field and the type of atomic nuclei being studied. In the ^1^H NMR experiment conducted on the soil samples, radiofrequency pulses at a frequency of 20 MHz were applied, causing the nuclear magnetization of hydrogen atoms in the sample to flip. Under a strong external magnetic field generated by permanent magnets, these magnetic moments of atomic nuclei add up, resulting in the macroscopic magnetization of the sample. After the radiofrequency pulse (RF) is emitted, the atomic nuclei in the sample return to their initial state of magnetic equilibrium. During this process, these nuclei generate a free induction decay signal (FID), which is detected by the NMR detector. The FID (free induction decay) signal from liquid water is faster than the FID signal from ice due to the difference in the mobility of molecules between these two states of matter. This allows for the assessment of changes in unfrozen water content in a freezing/thawing system.

##### NMR Procedure

The research procedure involved filling a glass NMR tube to a height of 1 cm with a paste-like soil sample and placing it in the magnetic chamber of the apparatus at a temperature of 25 °C. To be apprised of the detailed methodology, refer to our previous work [12]. This height allowed the sample to remain within the magnetic field interaction range, was consistent with the manufacturer’s data, and was experimentally confirmed. Reading the FID (free induction decay) values at room temperature allowed for the monitoring of the paramagnetic curve progression in the positive temperature range. Subsequently, the sample placed in the apparatus chamber was frozen to a temperature of −40 °C at a rate of approximately 2.5 K/min, and then it was sequentially thawed to temperatures of −32 °C, −23 °C, −14 °C, −9 °C, −5 °C, −3 °C, −1 °C, 0 °C, 1 °C, 3 °C, 5 °C, and 15 °C. After reaching the desired temperature, the sample was stabilized for at least 10 min before the FID values were read. In the case of phase transition temperatures, FID signal stabilization took longer, averaging around 2 h.

To eliminate the noise impact caused by electromagnetic field inhomogeneity, the unfrozen water content at a given negative temperature was calculated by averaging the FID signal values from five independent measurements.

##### Calculation of Unfrozen Water Content Based on NMR Technique

The equations used to calculate unfrozen water content may vary depending on the type of sample, NMR instrument, and experimental conditions. Additionally, this process can be more complex for heterogeneous samples or those containing multiple components. In this study, the unfrozen water content was calculated in several ways using Equation (1)

(1) $$w_{u}=\frac{{SI}_{FID}(T<0\;{}^{\circ}C)\cdot w_{n}}{{SI}_{FIDreference}^{1,2,3,4}}$$

where w_u_ is the unfrozen water content (% of soil dry weight), SI_FID_ is the signal intensity of free inductive decay at a given temperature, and w_n_ is the natural water content of the soil (% soil dry matter). (1) The highest signal value for a given sample above 0 °C was determined (a new insight uncovered in this study), (2) T = 0 °C [19]. (3) The reference signal values for each of the negative temperatures were distinct. This value was determined based on a function created individually for each sample. The function was constructed using changes in the FID signal intensity at temperatures above 0 °C [21], (4) T = 5 °C [20].

#### 2.2.3. DSC and Calculation of Unfrozen Water Content Based on DSC Technique

The unfrozen water content in copper-rich bentonites was determined using the DSC method in order to compare the results with the unfrozen water content determined using the NMR method. The experimentation was conducted using a DSC Q200 instrument (New Castle, DE, USA) manufactured by TA Instruments, coupled with a liquid nitrogen cooling system denoted as RSC 90. The samples were subjected to a cooling process carried out at a rate of 2.5 Kelvin per minute until they reached a temperature of −90 °C. Subsequently, they were heated at a rate of 5 Kelvin per minute until they reached 20 °C. The freezing and thawing rates were established experimentally [30]. To account for the potential occurrence of overcooling, the analysis focused on the heating thermograms. The unfrozen water content at a specific temperature was determined using Equation (2)

(2) $$w_{u}\left(T_{i}\right)=w-\sum_{j=i}^{n}\frac{100\cdot q(T_{j})\cdot ∆T_{j}}{L(T_{j})\cdot m_{s}}$$

where $w_{u}\left(T_{i}\right)$ is the unfrozen water content at temperature $T_{i}$ as a percentage of dry mass, w is the water content expressed as a percentage of dry mass, $m_{s}$ is the mass of dry soil in the sample (gram), and $L\left(T_{j}\right)$ is the latent heat of fusion of ice at temperature $T_{j}$ calculated according to the empirical equation $L\left(T\right)=7.3\cdot T+334$.

## 3. Results and Discussion

### 3.1. Optimization of the Method for Determining Changes in Unfrozen Water Content Using the ^1^H NMR Technique

Based on the changes in the intensity of the decaying signal of the free precession of hydrogen ions (FID) at specific temperatures below zero, the unfrozen water content was calculated using Formula (1). In Formula (1), we propose various methods for assessing the value of the reference FID signal (the signal for clay with natural water content). Generally, in the range of positive temperatures, the water content of clay does not change, and yet the FID signal begins to decrease. It is likely that the observed FID signal represents the convolution of the FID signal from the sample and instrument noise. Materials with even a slight paramagnetic property, such as water, exhibit a decrease in signal amplitudes as temperature increases [31]. 

The authors of [21,22], assuming the existence of a paramagnetic regression line in Equation (1), suggested calculating the reference FID signal value for each negative temperature individually. This calculation is based on a linear function derived from the changes in FID signal intensity at temperatures ≥ 0 °C. Other researchers suggested adopting the reference FID signal value at 0 °C [19] and 5 °C [20]. The authors approximated the FID signal value based on the results of soil samples devoid of ice at temperatures of 5 °C and 10 °C.

It remains unclear which of the above-mentioned methods should be applied for assessing the reference value of the FID signal in soil studies. Each of the studies utilized a different research material: coal [21], highly organic turf soil [22], kaolinite, pyrophyllite, volcanic ash, soft mudstone (predominantly illite and chlorite) [19], kaolinite, and bentonite (STx-1b) [20]. Furthermore, the research was conducted using different pieces of equipment and under varying experimental conditions. The assumption of a linear paramagnetic regression line may not entirely hold true in the case of soil studies, as soils constitute heterogeneous materials. The Curie law is not commonly applied at negative temperatures, and signal properties and behavior depend on numerous factors, such as sample type, interactions, temperature, and concentration. The FID signal in NMR studies is generated by the rotation of nuclear magnetic moments in response to a magnetic pulse. However, these magnetic moments may also be subject to relaxation, leading to a decrease in signal intensity over time. Bloembergen and Morgan [32] mentioned the dipole–dipole mechanism, which can influence spin relaxation times T1 and T2, directly affecting the length and shape of the FID signal. At positive temperatures, this relaxation can occur faster than at lower temperatures, potentially resulting in faster signal decay. Additionally, it cannot be ruled out that, at positive temperatures, the nuclear atoms in the sample may be thermally more active, and interactions with the environment may influence the behavior of magnetic moments, contributing to faster signal decay.

**Figure 2:**
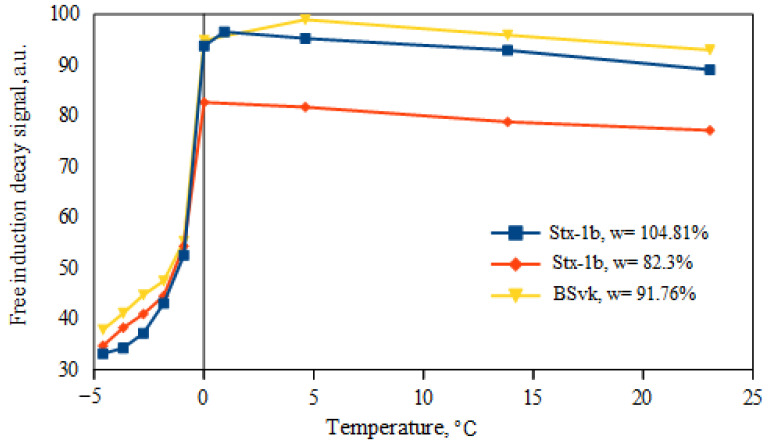
The relationship between FID signal values and temperature for source clays from the US (Stx-1b) and Slovakia (BSvk).

In this paper, the optimization of Equation (1), which defines the unfrozen water content in soils, was performed. This process entails taking the highest free induction decay (FID) signal value within the range of positive temperatures as the reference FID value, individually determined for each soil sample. It is most likely the case that the signal reaches its highest value at the thawing point. Soils, being heterogeneous materials, may not thaw at 0 °C [33]. In this study, it was observed that the temperature at which the maximum FID signal is achieved (the reference signal) may vary for each soil sample (Figure 2). In these experiments, the temperatures at which the FID values were highest were 0 °C, 1 °C, and 5 °C, respectively. Similar observations of the highest FID signal values at temperatures of 0 °C [19] and 5 °C [20] have been reported in studies conducted on soils by other authors.

The validation of the method was conducted by testing 21 samples of source clays: BSvk (a monoionic form of calcium bentonite from Slovakia) and Stx-1b (a monoionic form of calcium bentonite from the USA). The authors of [34] emphasize that validation of the adopted measurement method is particularly important when studying thermal characteristics in physical systems. To achieve this, the results depicting changes in unfrozen water content were compared at specific temperatures––namely, −32 °C, −23 °C, −14 °C, −7 °C, −5 °C, and −3 °C, as determined using the optimized NMR method––with the results from independent calorimetric tests. Furthermore, the optimized results for changes in unfrozen water content were compared to those obtained through procedures proposed by other researchers [19,20,21,22]. The outcomes obtained are presented in Table 2.

High agreement in results was achieved (Table 2) between the unfrozen water content determined for source clays using the DSC and NMR methods. The unfrozen water content results obtained through the NMR method based on the procedure by Li et al. [21] showed the largest deviations from the calorimetric results. The results closest to the calorimetric data were obtained using the optimized NMR method. Considering that, in the tested soils, the majority of samples exhibited the highest FID signal value at 5 °C, the optimized method’s results exhibit the strongest correlation with the findings of Kruse et al. [20].

### 3.2. Comparison of Changes in Unfrozen Water Content Determined via DSC with those Determined via NMR in Copper-Enriched Bentonites

Research on determining changes in the unfrozen water content using NMR and calorimetric methods conducted on source clays allowed for the assumption of the applicability of the optimized NMR method for studying copper-enriched clays.

Previous studies by the authors conducted on copper-enriched bentonites suggested the possibility of the occurrence of the chemical shift phenomenon when using the NMR method [12]. To confirm or refute the observed phenomenon, studies were conducted on a wider range of research materials covering a broader range of copper concentrations. Subsequently, utilizing the optimized NMR method, changes in the unfrozen water content in copper-enriched bentonites were assessed and compared with the results of calorimetric studies. The results were compiled in Table 3.

In each of the examined clays, the unfrozen water content determined using the NMR method was found to be lower than that determined using the calorimetric method. The observed phenomenon may result from the chemical shift in water molecules participating in the hydration process of paramagnetic ions, making them only partially visible via NMR [12]. Copper naturally possesses an unpaired electron, making it inherently paramagnetic. However, for better stability, one electron from the s orbital shifts to the d orbital. In metallic copper, the odd electron is shared in the pool of electrons forming metallic bonds, thus making the metal diamagnetic, while Cu^2+^ salts are paramagnetic [35]. In their studies, the cited researchers observed that two or three layers of water containing paramagnetic Cu(H_2_O)_6_^2+^ adsorbed on silica gel were strongly immobilized [36]. The observed Electron Spin Resonance (ESR) spectra were attributed to copper–water complexes in the aqueous solution, and their shape was closely related to changes in temperature. According to the authors, two relaxation mechanisms account for the observed phenomenon: (I) Spin rotation, independent of the applied field, and nuclear quantum spin number. Increasing the temperature leads to the broadening of individual transitions (which depends on the reciprocal of the Debye reorientational correlation time). (II) The modulation of anisotropic g and A tensors, dependent on the applied field, and the nuclear quantum spin number. The mechanism is controlled by the correlation time, which is associated with the movement of the Jahn–Teller distortion axis. This phenomenon is characteristic of certain types of molecules and ions in chemical systems. When this distortion axis changes its position, it affects the width of the magnetic resonance spectrum. Its value and characteristics change depending on certain parameters, such as temperature or other properties of the molecules, such as their mobility in a given system. As a result, the width of each individual transition in the magnetic resonance spectrum decreases with an increasing temperature as a consequence of the dynamic process of Jahn-Teller distortion axis correlation [36]. The research conducted in this study confirms there is a decrease in differences between the content of unfrozen water determined using DSC and NMR methods with an increasing temperature. 

At low temperatures (−32 °C and −23 °C), where the interaction with the clay surface has the greatest impact on the changes in unfrozen water content [12,13], it was observed that the differences between the unfrozen water content determined using DSC and NMR methods are higher with an increasing molar concentration of the solution (0.1–1 M). In the same direction, the stability of copper (II) ions decreases, likely due to increased competition from ions dissolved in the solution that complex with the surface of the clay mineral as the ionic strength of the solution increases [37]. 

The research results (Table 3) have shown that the differences between the unfrozen water content determined using the DSC and NMR methods vary depending on the molar concentration, copper ion concentration, and temperature. The statistical significance of these parameters will be assessed below due to the complexity of the issue. Different discrepancies in unfrozen water content at various temperatures may also indicate their connection with the physicochemical properties of the surface interacting with copper ions [13].

### 3.3. Analysis of the Relationship between Unfrozen Water Content, as Determined via DSC and NMR Techniques, and the Copper Concentration and Physicochemical Parameters of Bentonites

**Figure 3:**
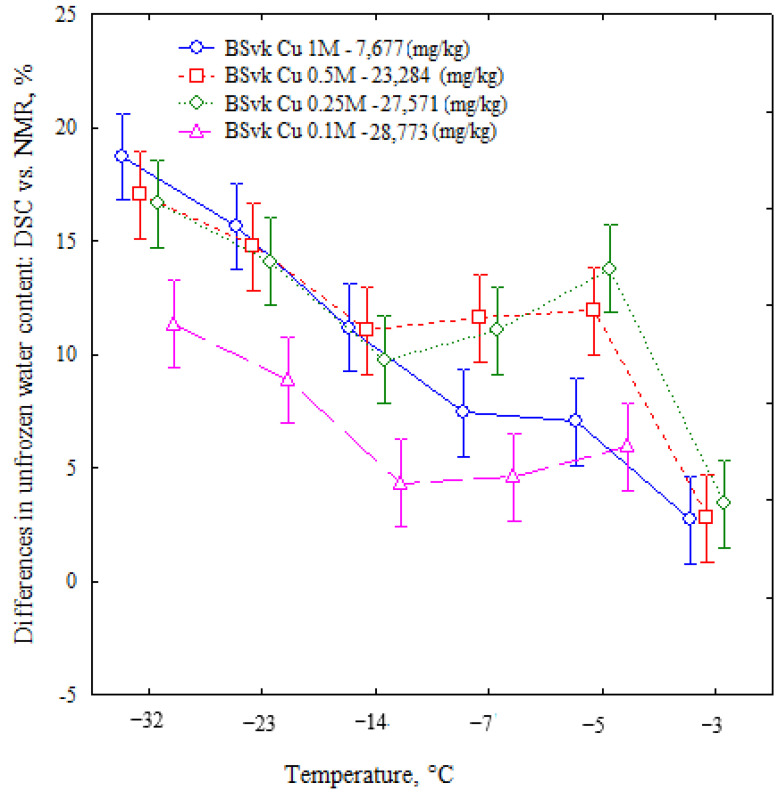
Differences in unfrozen water content regarding DSC vs. NMR determined for Slovak bentonite with different copper ion concentrations.

The unfrozen water content determined using the NMR method depends on changes in temperature and the molar concentration of a solution (Table 3). These dependencies were also confirmed in earlier studies by the cited authors regarding unfrozen water content determined using the DSC method [13]. The differences in unfrozen water content determined using calorimetric and magnetic resonance methods also appear to be related to temperature and molar concentration or copper ion concentration (Figure 3).

The observed relationships (Figure 3) have been statistically confirmed (Table 4).

The statistical analysis showed that the difference in unfrozen water content determined using the DSC and NMR methods was smaller with a decreasing molar concentration of the solution (*p* < 0.05) (at t = −32 °C (R = −0.83); −23 °C (R = −0.83); −14 °C (R = −0.75)) (Table 4). At temperatures of −7 °C and −5 °C, the differences ceased being significant. This could result from the dominance of cation–water interactions over water–water interactions at lower temperatures. Due to the cation–water interactions at low temperatures, tightly bound water forms on the surface of montmorillonite [38]. The biggest differences between the unfrozen water content determined using the DSC and NMR methods were observed in 1M bentonite. This likely indicates that this bentonite had the highest mobility of copper ions. Due to the higher mobility of copper, a larger portion of water molecules participate in the hydration of copper ions, becoming only partially visible via nuclear magnetic resonance [26]. The smaller differences in unfrozen water content between the DSC and NMR methods in the 0.1–0.5 M bentonites can be attributed to the lower mobility of Cu ions, some of which have been bound to form a new mineral phase in the form of atacamite [13]. The lowest differences in the 0.1 M bentonite are likely related to their being in the least acidic environment, in which Cu ions undergo hydration to a lesser extent than in 0.25 M and 0.5 M bentonites.

**Figure 4:**
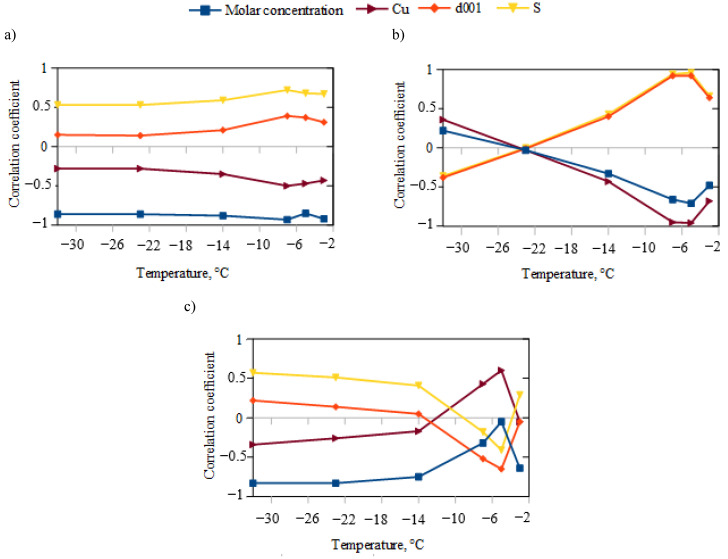
Correlation coefficient between unfrozen water content determined using various methods and the physicochemical properties of clay: (**a**) DSC method; (**b**) NMR method; (**c**) differences between DSC and NMR methods. Soil parameters tested: molar concentration—1; 0.5; 0.25; and 0.1 M of copper (II) chloride used for ion exchange; Cu—copper concentration (mass of dry weight) (ICP-OES method); d_001_—inter-packet distance (XRD method); S—specific surface (water sorption test method).

Certain relationships were observed between the unfrozen water content determined using the NMR and DSC methods and the physicochemical parameters of the clays (Figure 4).

Compared to the unfrozen water content determined using the DSC method, the unfrozen water content determined using the NMR method is less dependent on the molar concentration (NMR (R_t = −7°C_ = −0.66; R_t = −5°C_ = −0.71) vs. (R_t = −7°C_ = −0.93; R_t = −5°C_ = −0.85) and more dependent on the specific surface area (NMR (R_t = −7°C_ = 0.94; R_t = −5°C_ = 0.96) vs. DSC (R_t = −7°C_ = 0.72; R_t = −5°C_ = 0.68)). A statistically significant relationship was observed between the unfrozen water content determined using the NMR method and the concentration of copper ions (R_t = −7°C_ = −0.95; R_t = −5°C_ = −0.96), specific surface area (R_t = −7°C_ = 0.94; R_t = −5°C_ = 0.96), and interplanar distance (R_t = −7°C_ = 0.92; R_t = −5°C_ = 0.92). At a temperature of −3 °C, the soil-thawing process is most dynamic, and changes in unfrozen water content measured using the NMR method are related to molar concentration (R = −0.64), copper ion concentration (R = −0.68), specific surface area (R = 0.66), and interplanar distance (R = 0.64). High correlations between differences in unfrozen water content (DSC vs. NMR) and molar concentration as well as specific surface area at low temperatures may confirm the dominance of cation–water reactions at temperatures ≤ −14 °C (Figure 4) [38]. At temperatures of −7 °C and −5 °C, the differences in unfrozen water content increase with the rise in copper ion concentration and the decrease in interlayer spacing. This phenomenon may support the findings of Wang et al. [38], indicating that at higher water content, water–water interactions play a dominant role. Copper ions become less detectable via NMR as the concentration of free Cu ions increases, accompanied by a decrease in the interlayer spacing from which they are likely released. Consequently, water molecules hydrating copper ions become partially invisible in NMR examination [39].

## 4. Conclusions

1. The unfrozen water content in model bentonites determined using the optimized NMR method showed the best correlation with the DSC results. The utilization of the Bruker minispec mq20 instrument enabled rapid and efficient measurement of unfrozen water content changes during continuous temperature variation in water–clay systems.
2. The unfrozen water content determined using the NMR method in bentonites contaminated with Cu ions was found to be approximately 2–18% lower compared to the results obtained via DSC.
3. Differences in unfrozen water content determined using the DSC and NMR methods are statistically significant and depend on molar concentration, copper ion concentration, and temperature, as confirmed via ANOVA analysis.
4. As the temperature increases, the differences in unfrozen water content between the DSC and NMR methods in bentonites contaminated with copper ions decrease in the temperature range from −32 °C to −2 °C. It is likely that at temperatures ≤ −14 °C, copper–water interactions are more predominant than water–water interactions.
5. The largest differences in unfrozen water content (DSC vs. NMR) occur at low temperatures (≤−14 °C), especially at higher molar concentrations. Copper ions appear to be less mobile at lower molar concentrations (≤0.5M), possibly due to the weakly acidic environment and the binding of some copper ions and their subsequent transformation into a new mineral phase (atacamite). As the ionic strength of the solution increases, copper ion mobility rises, and water molecules hydrating copper ions become partially visible via NMR.

In the context of potentially toxic metals, it is recommended to conduct calorimetric studies to investigate changes in unfrozen water content in contaminated clays. Further NMR research can focus on identifying the types of metals that influence free induction decay signals under different physicochemical conditions.

## Figures and Tables

**Table 1:** Physicochemical properties of the tested bentonites.

	Stx-1b	BSvk	BSvk Cu			
			1 M	0.5 M	0.25 M	0.1 M
Copper ^&^	8.97 ± 0.13	6.28 ± 0.06	7677 ± 70	28,773 ± 162	27,571 ± 90	23,284 ± 121
Mineral composition ^+^	75% Sm 20% Op 5% Qz	92% Sm 5% Qz 3% Bt	92% Sm 5% Qz 3% Bt	86% Sm 5% Qz 3% Bt 6% Atc	87% Sm 5% Qz 3% Bt 5% Atc	89% Sm 5% Qz 3% Bt 3% Atc
d_001_^+^	14.87	14.88	12.48	12.31	12.34	12.38
Clay *	18.50	19.80	20.50	16.00	18.50	18.00
Silt *	81.50	80.20	79.50	84.00	81.50	82.00
Specific surface area ^#^	568	671	460	203	189	183

^&^ total content of copper in the dry clay matrix determined using ICP-OES (mg/kg). ^+^ mineral composition and interplanar distance determined using XRD method (Å); ± standard deviation; Sm—smectite-, Op—opal-, Qz—quartz-, Bt—biotite-, and Atc—atacamite-group minerals. * clay (d ≤ 0.002 mm) and silt (0.002 mm < d < 0.063 mm) content determined using laser diffraction method. ^#^ specific surface area determined using water sorption test method (m^2^/g) [27].

**Table 2:** Variations in unfrozen water content determined using NMR and DSC methods, along with the disparities between the results at specific sub-zero temperatures.

Stx−1b							
Temperature	DSC	NMR *			Difference DSC/NMR		
		(1) in This Study	(2) acc. Akagawa (2012) [19]	(3) acc. Li et al. (2021) [21]	(1)	(2)	(3)
−32 °C	21.08 ± 1.04	20.53 ± 1.43	21.25 ± 0.42	17.31 ± 1.66	0.55	−0.17	3.77
−23 °C	22.60 ± 1.10	23.14 ± 2.25	23.12 ± 0.26	20.25 ± 1.10	−0.54	−0.52	2.34
−14 °C	26.37 ± 1.07	26.80 ± 2.63	26.78 ± 0.43	24.40 ± 1.02	−0.42	−0.41	1.97
−7 °C	32.91 ± 1.37	32.73 ± 2.00	30.39 ± 1.11	30.73 ± 2.15	0.18	2.52	2.18
−5 °C	35.62 ± 1.63	35.86 ± 3.46	35.86 ± 1.24	33.95 ± 1.18	−0.24	−0.24	1.66
−3 °C	40.14 ± 2.28	40.45 ± 3.06	40.48 ± 1.25	38.66 ± 2.39	−0.30	−0.34	1.48
**BSvk**							
−32 °C	23.51 ± 1.71	19.75 ± 0.35	20.23 ± 0.59	17.97 ± 0.96	3.76	3.28	5.54
−23 °C	24.23 ± 0.85	23.84 ± 0.79	24.41 ± 0.36	22.26 ± 1.62	0.39	−0.18	1.97
−14 °C	27.03 ± 0.53	26.62 ± 1.22	27.25 ± 0.67	25.45 ± 1.87	0.41	−0.22	1.58
−7 °C	32.20 ± 1.23	33.71 ± 1.77	34.50 ± 1.09	32.81 ± 2.31	−1.51	−2.30	−0.61
−5 °C	36.96 ± 3.16	37.26 ± 2.14	38.13 ± 1.41	36.45 ± 2.65	−0.30	−1.17	0.51
−3 °C	39.05 ± 1.55	45.10 ± 3.25	46.17 ± 2.97	44.33 ± 3.49	−6.05	−7.12	−5.28

Stx-1b; BSvk—source clays (bentonite from the USA and Slovakia) ± standard deviation. * acc. Equation (1). The reference signal was taken as (1) the highest signal value for a given sample above 0 °C, (2) signal at 0 °C, and (3) the signal value for each of the negative temperatures was determined based on a function.

**Table 3:** The results of changes in unfrozen water content determined using the optimized NMR method and calorimetric method at specific negative temperatures in bentonites with varying copper ion concentrations.

T	DSC	NMR *	Difference DSC/NMR	T	DSC	NMR *	Difference DSC/NMR
**BSvk 1 M**				**BSvk 0.5 M**			
−32 °C	22.43 ± 1.66	4.26 ± 0.94	18.17	−32 °C	23.03 ± 2.32	5.43 ± 1.00	17.60
−23 °C	22.43 ± 1.66	7.48 ± 0.53	14.95	−23 °C	22.47 ± 1.45	7.74 ± 0.51	14.73
−14 °C	24.78 ± 2.05	14.6 ± 1.75	10.18	−14 °C	24.16 ± 1.60	13.56 ± 1.69	10.60
−7 °C	31.95 ± 1.84	24.97 ± 0.94	6.98	−7 °C	28.25 ± 1.53	17.67 ± 1.30	10.58
−5 °C	36.00 ± 2.36	30.23 ± 0.81	5.77	−5 °C	32.79 ± 1.56	28.08 ± 1.70	4.71
−3 °C	38.04 ± 1.72	35.28 ± 0.71	2.76	−3 °C	34.40 ± 1.75	32.76 ± 2.36	1.64
**BSvk 0.25 M**				**BSvk 0.1 M**			
−32 °C	20.04 ± 1.53	4.47 ± 0.22	15.57	−32 °C	17.51 ± 1.38	5.20 ± 0.56	12.31
−23 °C	20.27 ± 1.18	7.04 ± 0.38	13.23	−23 °C	17.51 ± 1.38	7.67 ± 0.27	9.84
−14 °C	22.21 ± 0.68	12.9 ± 1.04	9.31	−14 °C	18.95 ± 1.51	13.57 ± 0.6	5.38
−7 °C	28.84 ± 0.51	17.73 ± 0.58	11.11	−7 °C	23.81 ± 1.21	18.79 ± 1.43	5.02
−5 °C	32.90 ± 2.68	27.56 ± 0.66	5.34	−5 °C	28.73 ± 0.69	28.93 ± 1.62	−0.20
−3 °C	34.56 ± 0.47	31.29 ± 1.44	3.27	−3 °C	29.10 ± 0.95	33.04 ± 1.39	−3.94

* acc. Equation (1). The reference signal was taken as (1) the highest signal value for a given sample above 0 °C, ± standard deviation; BSvk—bentonite from Slovakia after saturation of 1–0.1 M CuCl_2_^&^; ^&^ samples were rinsed from free chloride ions. Copper concentrations were 7677; 28,773; 27,571; and 23,284 (mg/kg), respectively.

**Table 4:** Univariate tests of significance (ANOVA) of the temperature, molar or copper concentrations, and their interactions for the unfrozen water content and differences between unfrozen water content determined using DSC and NMR techniques.

	Sum of Squares	Degrees of Freedom	Mean Square	F-Test Value	*p*-Value	Significance
Dependent variable: The unfrozen water content determined using NMR technique						
Intercept	21,131.04	1	21,131.04	16,939.65	0.0000	
Temperature (T)	6756.04	5	1351.21	1083.19	0.0000	***
Molar (M) or copper concentration (Cu)	149.69	3	49.90	40.00	0.0000	***
T · M or T · Cu	167.67	15	11.18	8.96	0.0000	***
Error	59.88	48	1.25			
Dependent variable: Differences between unfrozen water content determined using DSC and NMR techniques						
Intercept	6691.42	1	6691.42	2431.10	0.0000	
Temperature (T)	1511.15	5	302.23	109.80	0.0000	***
Molar (M) or copper concentration (Cu)	497.38	3	165.79	60.24	0.0000	***
T · M or T · Cu	112.18	15	7.48	2.72	0.0044	**
Error	132.12	48	2.75			

Temperature: −32 °C, −23 °C, −14 °C, −9 °C, −5 °C, and −3 °C. Molar concentrations: 1 M, 0.5 M, 0.25 M, and 0.1 M. Copper concentrations: 7677, 23,284, 27,571, and 28,773 (mg/kg). Significant at the *** 0.001 and ** 0.01 probability levels.

## Data Availability

Data are contained within the article.
